# Development of a variant of dinutuximab with enhanced antitumor efficacy and reduced induction of neuropathic pain

**DOI:** 10.1002/2211-5463.13464

**Published:** 2022-08-18

**Authors:** Xin‐Yuan Liu, Yi‐Li Chen, Guo‐Jian Liu, Xiang‐Nan Deng, Yue Cui, Jie Tan, Xing‐Chen Dong, Hua‐Ying Li, Gan‐Jun Chen, Zhi‐Min Ou, Chun‐He Wang

**Affiliations:** ^1^ College of Pharmaceutical Science Zhejiang University of Technology Hangzhou China; ^2^ Department of Antibody Discovery Shanghai Mabstone Biotechonology, Ltd. China; ^3^ Department of Reasearch and Development Center Dartsbio Pharmaceuticals Ltd. Zhongshan China; ^4^ School of Chinese Materia Medica Nanjing University of Chinese Medicine China; ^5^ Biotherapeutics Discovery Research Center, Shanghai Institute of Materia Medica Chinese Academy of Sciences Shanghai China; ^6^ University of Chinese Academy of Sciences Beijing China; ^7^ Faculty of Life Science and Biopharmaceutics Shenyang Pharmaceutical University China

**Keywords:** ADCC, affinity maturation, antibody engineering, anti‐GD2 antibodies, engineered Fc domain, humanized

## Abstract

Dinutuximab (ch14.18) was the first approved monoclonal antibody against the tumor‐associated antigen disialoganglioside GD2. Despite its success in treating neuroblastoma (NB), it triggers a significant amount of neuropathic pain in patients, possibly through complement‐dependent cytotoxicity (CDC). We hypothesized that modifying ch14.18 using antibody engineering techniques, such as humanization, affinity maturation, and Fc engineering, may enable the development of next‐generation GD2‐specific antibodies with reduced neuropathic pain and enhanced antitumor activity. In this study we developed the H3‐16 IgG1m4 antibody from ch14.18 IgG1. H3‐16 IgG1m4 exhibited enhanced binding activity to GD2 molecules and GD2‐positive cell lines as revealed by ELISA, and its cross‐binding activity to other gangliosides was not altered. The CDC activity of H3‐16 IgG1m4 was decreased, and the antibody‐dependent cellular cytotoxicity (ADCC) activity was enhanced. The pain response after H3‐16 IgG1m4 antibody administration was also reduced, as demonstrated using the von Frey test in Sprague–Dawley (SD) rats. In summary, H3‐16 IgG1m4 may have potential as a monoclonal antibody with reduced side effects.

## Graphical abstract

The anti‐disialoganglioside GD2 monoclonal antibody dinutuximab is an important option for the treatment of high‐risk neuroblastoma. We engineered dinutuximab through humanization, affinity maturation, and Fc mutation to obtain a promising preclinical agent. The engineered anti‐GD2 mAb H3‐16 IgG1m4 showed enhanced antitumor activity *in vitro* and reduced neuropathic pain side effects *in vivo*.

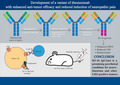

**Abbreviations**
ADA: anti‐drug antibody
ADCC: antibody‐dependent cellular cytotoxicity
BLI: bio‐layer interferometry
CDC: complement‐dependent cytotoxicity
CDR3: complementarity determining region 3
*EC*_*50*_
: half‐maximal effective concentration
EFS: event‐free survival
ELISA: enzyme‐linked immunosorbent assay
FcγR: Fc gamma receptors
FR: framework region
GM‐CSF: granulocyte‐macrophage colony‐stimulating factor
HEK: human embryonic kidney
HER2: human epidermal growth factor receptor type 2
HPLC‐SEC: high‐performance size‐exclusion chromatography
IgG1: immunoglobulin G1
IL‐2: interleukin‐2
NB: neuroblastoma
OS: overall survival
SDS/PAGE: sodium dodecyl sulfate polyacrylamide gel electrophoresis
VH: heavy‐chain variable domain
VL: light‐chain variable domain

Neuroblastoma (NB) is one of the most common solid tumors in children, comprising 8–10% of pediatric malignancies. Eighteen months is the median age of diagnosis of NB in patients, and 40% of patients are diagnosed in infancy and 90% are younger than 10 years old [1]. Primary tumors can occur anywhere in the sympathetic nervous system, but is most common in the adrenal medulla and paraspinal ganglia. In all NB patients, greater than 50% are diagnosed with metastatic disease typically located in the regional lymph nodes, bone marrow, bones, liver, and skin tissues [2]. According to the International Neuroblastoma Risk Group (INRG) risk classification criteria, NB patients are divided into four stages: very‐low‐risk, low‐risk, intermediate‐risk, and high‐risk groups [3]. Among the patients in the high‐risk group, even after intensive chemotherapy, surgical resection, radiotherapy, or myeloablative hematopoietic stem cell transplantation, the 5‐year event‐free survival (EFS) rate is still between 30% and 50%. In addition, some patients can be classified as ultra‐high‐risk groups, and their 5‐year EFS and overall survival (OS) rates are both 0% [4]. Therefore, discovering novel NB treatments and improving existing NB treatments are of great clinical significance.

There are cases of spontaneous tumor regression in NB patients [5], and it is believed that NB has a unique immune mechanism. Thus, tumor‐specific antigens have been explored, and disialoganglioside GD2 was discovered on most NB cells [6]. In normal tissues, only low levels of GD2 were identified in nerve fibers, melanocytes, and neurons [7]. In clinical trials, both murine anti‐GD2 monoclonal antibodies (mAbs), 3F8 and 14.G2a, showed antitumor activity [8, 9] but also triggered intense neuropathic pain and other side effects in patients [10], limiting their therapeutic windows [11].

Several immunotherapies are being developed to increase the efficacy of GD2‐specific antibodies and reduce their side effects [12]. The antitumor mechanisms of antibodies targeting GD2 are that the antibodies directly induce cell death, antibody‐dependent cell‐mediated cytotoxicity (ADCC), and complement‐dependent cytotoxicity (CDC). It has been demonstrated that the ADCC effects play a key role against tumor cells [13], and toxicity may be partly due to the CDC effect against normal peripheral nerve fibers [14] and immunogenicity induced by nonhumanized antibodies. The application of humanized and CDC‐modified antibodies (hu3F8, hu14.18K322A) has improved outcomes, produced favorable pharmacokinetic characteristics, and reduced toxicity [15, 16, 17].

Dinutuximab (ch14.18) was the first chimeric GD2 mAb approved by the United States Federal Drug Administration (FDA) in 2015 to treat high‐risk NB patients. The efficacy of dinutuximab in combination with granulocyte‐macrophage colony‐stimulating factor (GM‐CSF), interleukin‐2 (IL‐2), and isotretinoin acid (RA) is significantly superior to that of RA alone [18]. Ch14.18 is a human and mouse chimeric antibody that originated from the murine antibody 14.G2a, but it still has the side effects of pain, hypotension, capillary leak syndrome, and hypersensitivity reactions. Humanized antibody hu14.18K322A shares the same IgG1‐κ as ch14.18, but the K322A mutation significantly reduces CDC activity [16], and clinical trials have shown that hu14.18K322A has less painful side effects [17]. In order to enhance the antitumor activity of the engineered antibody, hu14.18K322A was expressed in rat myeloma YB2/0 cells, and this expression system produced an antibody with low fucose content that enhanced the ADCC effect of the antibody. In the example of the anti‐HER2 antibody engineering [19], its modification of the Fc domain acquired an increased binding affinity to Fcγ receptors IIIA and enhanced the function of ADCC. In this study, we engineered ch14.18 with humanization, affinity maturation, and the Fc mutation [16, 19, 20, 21] to obtain anti‐GD2 mAb H3‐16 IgG1m4 with enhanced antitumor activity and reduced immunogenicity and neuropathic pain side effects.

## Materials and methods

### Cell lines

The human NB cell line, IMR‐32, and human glioblastoma cell line, U‐87 MG, were purchased from the BeNa Culture Collection (BNCC, China). Cells were cultured in a Roswell Park Memorial Institute (RPMI) 1640 medium supplemented with 10% fetal bovine serum (Gibco, Waltham, MA, USA) at 37 °C in a 5% CO_2_ incubator.

### Antibody expression and purification

The gene sequences encoding antibodies were respectively subcloned into mammalian expression plasmids (pEE12.4 or pEE6.4) that were a gift from Dr. Chun‐He Wang (Shanghai Institute of Materia Medica). We inserted the human antibody light chain constant region (CL) sequence into the pEE12.4 plasmid. After inserting the variable region sequences of the light chain (VL) into the pEE12.4, a complete antibody light chain expression plasmid was obtained. In the pEE6.4 plasmid, we embedded the sequence of heavy chain constant regions (CH) that contained human wildtype (WT) immunoglobulin G1 (IgG1). When we inserted the heavy chain variable region sequence (VH) into the pEE6.4, it was reconstituted into a complete antibody heavy chain expression plasmid. A recombinant mAb protein was produced by the transient transfection of suspension cells, HEK 293F, and the cells were cultured in OPM‐293 CD05 medium (OPM Biosciences, Shanghai, China) and incubated in a shaker incubator at 37 °C, 120 r.p.m., and 5% CO_2_. The two plasmids and polyethyleneimine (PEI) were premixed and added dropwise to the cell culture. After transfection, the cells were cultured in a shaker incubator for ~ 5 days, and the mature supernatant was collected. The antibodies were purified from cell culture supernatants using Protein‐A affinity column chromatography (BestChrom, Shanghai, China) and detected by size‐exclusion chromatography (SEC).

### Antibody engineering

Ch14.18 IgG1 is a human–mouse chimeric IgG1 antibody with kappa light chain. We successively constructed two mutant phage display libraries about ch14.18 IgG1 and screened them to obtain a humanized and affinity‐matured anti‐GD2 antibody H3‐16 IgG1. Ch14.18 IgG1 was humanized by framework shuffling [22]. This was followed by the random mutagenesis of heavy chain complementarity determining region 3 (CDR3) to increase its affinity for GD2 [23].

First, we downloaded the variable region sequences of VL and VH of the ch14.18 IgG1 antibody from the international immunogenetics information system (www.imgt.org) and used them as templates to synthesize the VH and VL genes. The human antibody framework genes were obtained from the open‐access gene bank of the human germline antibody. The murine framework regions (FRs) were randomly replaced by human FRs using PCR. The humanized VH and VL genes were cloned into the M13‐based phage expression vector and then electroporated into *Escherichia coli* XL1‐Blue to establish a humanized phage library. After two rounds of panning to enrich the phage clones binding to GD2, the eluted phages were screened using a filter lift. We obtained several humanized genetic combinations of the VH and VL regions from ch14.18 IgG1 by cloning and sequencing the single phage clones that can bind to GD2, and we constructed these regions into mammalian expression vectors. The vectors were transformed into the host cell, HEK 293F, and the antibodies were purified as described above. We selected a humanized antibody, 2T10 IgG1, that has similar antigen‐binding activity as ch14.18 IgG1 for subsequent modification.

Second, according to the VH sequence of the 2T10 IgG1 antibody, CDR3 random mutation primers were designed. Using PCR technology, the VH genes after the random mutation of amino acids in the CDR3 region were cloned into a phage vector carrying the VL gene of 2T10 IgG1. We electroporated the recombinant plasmid into competent XL1‐Blue and obtained an affinity‐matured phage library. After three rounds of panning to enrich the phage clones binding to GD2, the eluted phages were screened by the filter lift. We obtained several affinity‐matured antibodies. After screening for the antigen‐binding activity, we selected H3‐16 IgG1 to perform the following research.

Finally, the genes of VH and VL of H3‐16 IgG1 were grafted into a mutant human IgG1 (IgG1m4) that had amino acid mutations designed to prevent the activation of the complement cascade [16, 20] and to enhance the effect of ADCC [19, 21]. This antibody was numbered H3‐16 IgG1m4, and is studied in detail below.

### SDS/PAGE

Antibodies were expressed in the HEK 293F cells, and the purified products were analyzed using 10% SDS/PAGE to check their purity. We prepared the resolver solution and stacker solution according to the manufacturer's instructions (TGX FastCast Acrylamide Kit; Bio‐Rad, Hercules, CA, USA), assembled the electrophoresis device after gel polymerization, and filled the electrophoresis tank with a running buffer (24.77 mm of HTAM, 250.43 mm of glycine, and 3.47 mm of SDS). A reducing or nonreducing loading buffer (Beyotime, Shanghai, China) was added to 3 μg protein, and the mixture was heated at 100 °C for 10 min in order to obtain the heat‐denatured protein. The sample volume was 10 μL, and the Prestained Protein ladder (Thermo, Waltham, MA, USA) was utilized as the protein molar mass marker. First, we used a constant voltage of 80 V to run for 30 min and then increased the voltage to 120 V to run for ~ 40 min. After degumming, the gel was stained with Coomassie brilliant blue R‐250 for 60 min at room temperature (RT), followed by removal of the staining solution and then destaining overnight with a destaining solution. A gel imager (Bio‐Red ChemiDoc MP; Bio‐Rad) was used to photograph the gel, and the software used was image lab 5.2.1 (Bio‐Rad).

### HPLC‐SEC

When performing the HPLC‐SEC analysis, the solution of antibodies was centrifuged at 16 000 × ***g*** for 10 min, and only the first third of the solution was aspirated to prepare the test samples to avoid large protein aggregates. The antibody purity was detected using an Agilent 1260 HPLC system, and the absorbance at 280 nm was monitored. The system was formulated with a MabPac SEC‐1 (Thermo) size exclusion column, 4 × 300 mm, 5 μm, and 300 Å. The mobile phase contained 25.30 mm of NaH_2_PO_4_, 30.50 mm of Na_2_HPO_4_, and 300 mm of NaCl at a pH of 6.8. Samples with a concentration of 1 mg·mL^−1^ and a volume of 10 μL were run in the mobile phase for ~ 20 min. The flow rate was 0.2 mL·min^−1^, the maximum column pressure was 55 bar, and the column temperature was 25 °C. The percentage of monomers was calculated using the area normalization method, and the system software was lc1260 (Agilent Technologies, Palo Alto, CA, USA).

### ELISA of targeting GD2

The 96‐well ELISA plates (Greiner Bio‐One, Monroe, NC, USA) were coated by incubating 0.5 μg·mL^−1^ of GD2‐KLH (Elicityl‐oligotech, Clos, France) in PBS overnight at 4 °C. The next day, the plates were washed three times with wash buffer and blocked with 1% casein in the PBS for 1 h at 37 °C. The primary antibodies of ch14.18 IgG1, H3‐16 IgG1m4, or isotype IgG1 were prepared to 15 μg·mL^−1^ in 0.1% casein in the PBS, and subsequently, the samples were made into 11 serial three‐fold dilutions. After the blocking solution was discarded and the plates were washed, serial dilutions of the primary antibodies were added to the plates in duplicate and incubated at 37 °C for 1 h. The plates were washed six times with 0.1% PBS‐T (added Tween‐20 to PBS, v/v) and incubated with goat anti‐hIgG‐horseradish peroxidase (HRP; Abclonal, Wuhan, China) at 37 °C for 1 h. After being washed with 0.1% PBS‐T, the plates were developed and the absorbance was read at 450 nm using a SpectraMax M5e (Molecular Devices, San Jose, CA, USA) microplate spectrophotometer.

### ELISA for cross‐reactivities

GD2‐KLH, GD1a‐KLH, GD1b‐KLH, GD3‐KLH, GM1a‐KLH, GM1b‐KLH (Elicityl‐oligotech), and GM2‐KLH were coated on 96‐well ELISA plates (Greiner Bio‐One) at 30 μg·mL^−1^ in PBS. They were incubated at 4 °C overnight, and the wells were blocked with 1% casein in PBS at 120 μL per well for 1 h at 37 °C. Primary antibodies of anti‐GD2 were added at 10 μg·mL^−1^ in 0.01% casein. After incubation at 37 °C for 1 h and washing with 0.1% PBS‐T, goat anti‐hIgG‐HRP (Abclonal) was added, and the plates were incubated at 37 °C for 1 h. After washing, the chromogenic substrate was added, and the OD was read using a SpectraMax M5e microplate spectrophotometer (Molecular Devices) at 450 nm. The absorbance of the wells containing only the primary antibodies but no antigen was used as the background value. After excluding the background values, the cross‐reactivity of the anti‐GD2 mAbs was expressed as a percentage of binding to other gangliosides and GD2.

### Flow cytometry analysis

Flow cytometry was used to analyze the binding of H3‐16 IgG1m4 to GD2‐positive tumor cells *in vitro*. The NB cell line, IMR‐32 (3 × 10^5^ cells per well), was added to the 96‐well U‐bottom plates. Serial three‐fold dilutions of anti‐GD2 antibodies or an isotype control were prepared in the PBS and added to each well. The plates were incubated for 1 h at 4 °C. The cells were washed three times with PBS, followed by subsequent staining with P‐phycoerythrin (PE)‐conjugated mouse anti‐human IgG Fc (Biolegend, San Diego, CA, USA) in the dark at 4 °C for 0.5 h. After washing the plates, the cells were resuspended in 200 μL of PBS per well, and an aliquot of 1 × 10^4^ was analyzed on a CytoFLEX flow cytometer (Beckman Coulter, Indianapolis, IN, USA). The background control cells were prepared using the second antibody alone.

### Bio‐layer interferometry (BLI)

The affinities of the different Fc mutants for FcγRIIIa and FcγRIIa were determined using Fortebio Octet Red 96 (Fortebio, Fremont, CA, USA). Recombinant human CD16a‐F176, CD16a‐V176 (Acro Biosystems, Beijing, China), CD32a‐R167, and CD32a‐H167 (Novoprotein, Suzhou, China) all with the His‐tag were loaded on the Ni‐NTA biosensors (Fortebio) at 5 or 10 μg·mL^−1^ of the appropriate homemade buffers. H3‐16 IgG1 or H3‐16 IgG1m4 (mAbs) with the same Fab domains but different human Fc sequences was prepared to 750 or 15 μg·mL^−1^ in the same buffer as above, and subsequently the sample was made into seven serial two‐fold dilutions. After loading the ligand onto the biosensors, the association and dissociation were measured by transferring sensors to the antibody solution or the running buffer. Data were analyzed using the data analysis software fortebio.

### ADCC

The target cells of IMR‐32 and U‐87 MG were detached with pipettes, washed, and placed in 96‐well plates (Corning, Lowell, MA, USA) in R2 (RPMI 1640 medium supplemented with 2% fetal bovine serum) at approximately 3 × 10^4^ cells per well. Antibodies of H3‐16 IgG1m4 and ch14.18 IgG1 that were separately diluted in R2 were added, and the mixture was incubated at 37 °C for 1 h. The effector cells of human peripheral blood mononuclear cells (PBMCs) were isolated from the peripheral blood of healthy donors using Ficoll‐Paque PREMIUM (GE Healthcare, Chicago, IL, USA) and added into the plates (effect: target ratio of 20 : 1). The co‐culture was incubated in a 37 °C and 5% CO_2_ incubator for 18 h. The 50‐μL/well supernatant was then removed to determine the lactate dehydrogenase (LDH) activity using a Cytotoxicity Detection Kit (Roche, Basel, Switzerland) [24]. The absorbance was determined at 490 nm in a SpectraMax M5 (Molecular Devices). The percentage cytotoxicity was determined using the following equation:

$$\text{Cytotoxicity}\;\left(\%\right)=\frac{\text{experiment}-\text{effector}\&\text{target cell}\;\mathrm{mix}}{\text{high control}-\mathrm{low}\;\text{control}}\times 100\%.$$

### CD107a degranulation assay

For analysis of anti‐GD2 antibody‐activated NK cell activity, anti‐GD2 antibody and target cells (4 × 10^4^ cells per well) were added to 96‐well U‐bottom cell culture plates and then incubated for 1 h at 37 °C in a 5% CO_2_ cell culture incubator, followed by co‐culture with PBMCs (20 × 10^4^ cells per well, effect: target ratio of 5 : 1). The NK cell activation marker CD107a was assayed according to the method described by Alter et al. [25] and Gong et al. [26]. Target cells, antibodies, and PBMCs were co‐cultured for 2 h, followed by the addition of monensin (6 μg·mL^−1^, Yuanye, Shanghai, China) for a further 3.5 h. APC/Fire anti‐human CD107a antibody (1 : 133, Biolegend) was added and incubated for 0.5 h at 37 °C in a 5% CO_2_ cell culture incubator protected from light. Cell precipitate was collected by centrifugation at 300 × ***g*** for 5 min, then resuspended in PBS and incubated with PE anti‐human CD3 and FITC anti‐human CD56 antibodies (1 : 133, Biolegend) for 0.5 h at 4 °C in the dark. After washing by centrifugation and resuspension of the cell precipitate, the proportion of CD3^−^/CD56^+^/CD107a^+^ cells in the CD3^−^/CD56^+^ cell population was measured by flow cytometry (Beckman Coulter).

### CDC

For the CDC analysis, the target cells were incubated with ch14.18 IgG1 or H3‐16 IgG1m4 (1.5–15 000 ng·mL^−1^) in the presence of 2% normal human serum complement (Quidle, San Diego, CA, USA). After 18 h of incubation, the supernatant was collected to react with lactate dehydrogenase substrate (Roche), and the absorbance was measured using SpectraMax M5 (Molecular Devices). To calculate the percentage cytotoxicity, the following equation was used:

$$\text{Cytotoxicity}\;\left(\%\right)=\frac{\text{experiment}-\mathrm{no}\;\text{antibody control}}{\text{high control}-\mathrm{low}\;\text{control}}\times 100\%.$$

### von Frey

The experiments used 6–7‐week‐old (160–180 g) male Sprague–Dawley (SD) rats (specific‐pathogen‐free [SPF] animals) that were purchased from Beijing Vital River Laboratory Animal Technology. This study was conducted in strict accordance with the recommendations in the Guide for the Care and Use of Laboratory Animals of the National Institutes of Health (Bethesda, MD, USA). Tests were approved by the Institutional Animal Care and Use Committee (IACUC) of the Shanghai Institute of Materia Medica, Chinese Academy of Sciences (IACUC number: 2020‐03‐WCH‐04). All rats were kept in an SPF environment under a 12‐h day/night cycle with water and food available *ad libitum* and were allowed to recover from transport for 7 days prior to the experiments. The animals were placed in an individual test facility for 30–60 min until they stopped obvious exploratory behaviors and were in a quiet state. They were then acclimatized for 3 consecutive days. Furthermore, the animals were acclimated to the facility for at least 30 min before testing. Referring to the previous literature [27], the basal 50% probability mechanical withdrawal threshold for each rat was determined using the manual von Frey test. The animals were injected with an equal volume of antibody (1 mg·kg^−1^) solution or vehicle through the tail vein. The animals were tested 0.5, 1, 2, 3, 4, 5, 24, and 48 h after injection.

### Statistical analysis

An unpaired Student's *t*‐test or one‐way ANOVA was used for comparison between the two groups, and a two‐way ANOVA was used for comparisons between multiple groups. Data were represented as mean ± SEM and *P*‐values less than 0.05 were considered significant.

## Results

### H3‐16 IgG1m4 bound GD2 with increased affinity

Ch14.18 IgG1 is a human–mouse chimeric anti‐GD2 mAb that contains the constant region of human WT IgG1 and the variable region of the murine 14.18 antibody. Ch14.18 IgG1 was first humanized with framework shuffling, then affinity maturation was conducted with a random mutation phage display library. The humanized and affinity‐matured antibody was grafted into a mutated human IgG1 fragment (IgG1m4) to obtain H3‐16 IgG1m4.

**Fig. 1:**
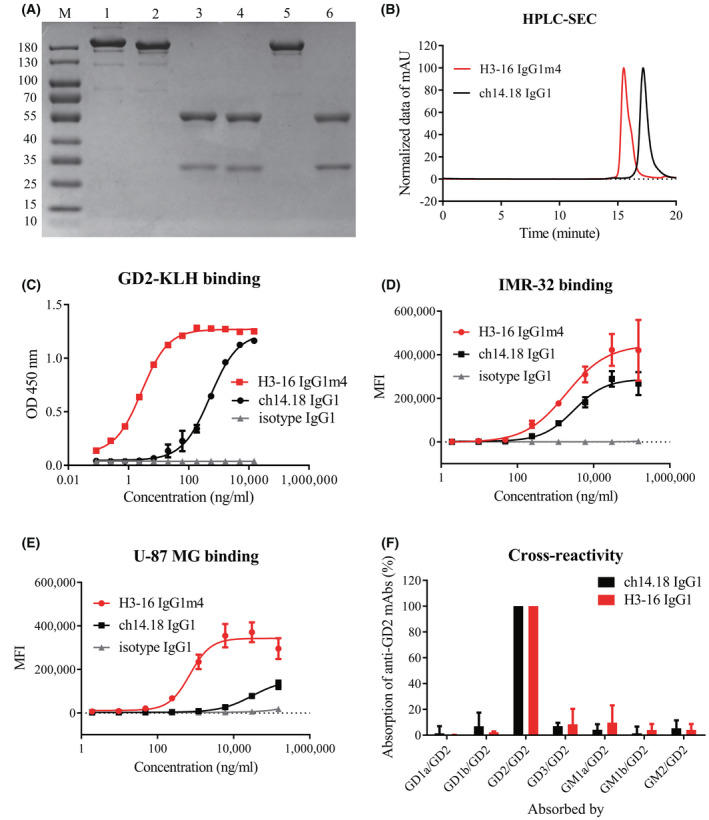
Binding analysis of H3‐16 IgG1m4 to GD2. (A) H3‐16 IgG1m4 was analyzed by reduced and nonreduced SDS/PAGE. M, molecular weight markers; 1, nonreduced ch14.18 IgG1; 2, nonreduced H3‐16 IgG1m4; 3, reduced ch14.18 IgG1; 4, reduced H3‐16 IgG1m4; 5, nonreduced isotype IgG1; 6, reduced isotype IgG1. (B) Purities of H3‐16 IgG1m4 were analyzed using size‐exclusion chromatography (SEC). The absorbance unit (AU) normalized to 0–100 and plotted with retention time. (C) Binding of H3‐16 IgG1m4 to plate‐bound GD2‐KLH. Serial dilutions of ch14.18 IgG1 (as a positive control), H3‐16 IgG1m4, or isotype IgG1 (as a negative control) were incubated with plate‐bound GD2‐KLH, and binding was assessed via anti‐human IgG ELISA. (D) Binding potency of H3‐16 IgG1m4 to the IMR‐32 cell line using flow cytometric. IMR‐32 was stained with ch14.18 IgG1, H3‐16 IgG1m4, or isotype IgG1 and PE mouse anti‐human IgG Fc, and the data of the median fluorescence intensity (MFI) were analyzed. (E) Binding potency of H3‐16 IgG1m4 to the U‐87 MG cell line using flow cytometric. (F) Absorption rates of the anti‐GD2 IgG1 antibodies by other gangliosides. In this study, H3‐16 and ch14.18 monoclonal antibodies with identical Fc sequences (IgG1) were incubated with plated‐bound GD2, GD1a, GD1b, GD3, GM1a, GM1b, or GM2, all coupled with KLH, and the binding was assessed via anti‐human IgG ELISA. Data are presented as mean ± SEM (*n* = 2) and evaluated using an unpaired Student's *t*‐test. [Colour figure can be viewed at wileyonlinelibrary.com]

The antibody proteins purified from the HEK 293F expression system were all of high purity (Fig. 1A). The results of HPLC‐SEC showed that, similar to ch14.18 IgG1, H3‐16 IgG1m4 also had excellent purity, and the purity was greater than 95% (Fig. 1B). The affinity of H3‐16 IgG1m4 binding to the GD2‐KLH molecule (*EC*_*50*_ = 0.018 nm) was greater than that of ch14.18 IgG1 (*EC*_*50*_ = 3.43 nm; Fig. 1C). For the NB cell line, IMR‐32, and the human glioblastoma cell line, U‐87 MG, the former also showed a higher binding affinity than the latter (Fig. 1D,E). Both antibodies showed low levels of cross‐reactivity with another ganglioside (Fig. 1F). Therefore, H3‐16 IgG1m4 had a higher affinity to ganglioside GD2 than ch14.18 IgG1.

### H3‐16 IgG1m4 reduced CDC and maintained the ADCC effects

**Fig. 2:**
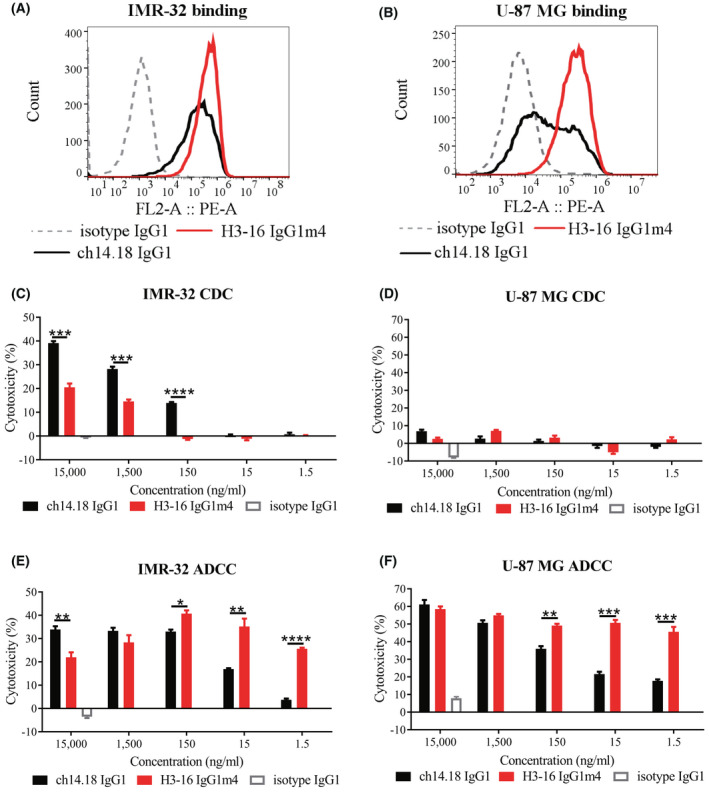
Anti‐GD2 monoclonal antibody potency was relative to H3‐16 IgG1m4 in antibody‐dependent cell‐mediated cytotoxicity (ADCC) and complement‐dependent cytotoxicity (CDC). (A) Flow cytometric analysis of the neuroblastoma cell line, IMR‐32, after staining of cells with ch14.18 IgG1, H3‐16 IgG1m4, and isotype IgG1. (B) Flow cytometric analysis of the glioblastoma cell line, U‐87 MG, after staining cells with antibodies. (C) Anti‐GD2 antibodies' potency in CDC against IMR‐32. An 18‐h LDH release assay after treating with ch14.18 IgG1 or H3‐16 IgG1m4 at a series of diluted concentrations from 15 to 0.0015 μg·mL^−1^. The isotype IgG1 antibody at a concentration of 15 μg·mL^−1^ served as a negative control. (D) Anti‐GD2 antibodies' potency in CDC against the U‐87 MG cells. (E) ADCC mediated by human PBMCs against the IMR‐32 cell line in the presence of ch14.18 IgG1 or H3‐16 IgG1m4 at a series of diluted concentrations from 15 to 0.0015 μg·mL^−1^. An 18‐h LDH release assay detected the percentage of cytotoxicity. The isotype IgG1 antibody at a concentration of 15 μg·mL^−1^ served as a negative control. (F) Anti‐GD2 antibodies' potency in ADCC against U‐87 MG. Data are presented as mean ± SEM (*n* = 3) and evaluated using an unpaired Student's *t*‐test (*P < 0.05, **P < 0.01, ***P < 0.001, and ****P < 0.0001). [Colour figure can be viewed at wileyonlinelibrary.com]

The flow cytometry results showed that H3‐16 IgG1m4 bound to the IMR‐32 and U‐87 MG cells more efficiently than ch14.18 IgG1 (Fig. 2A,B). Under our testing conditions, the complement‐related cell lysis of IMR‐32 by H3‐16 IgG1m4 was lower than that by ch14.18 IgG1 (Fig. 2C). However, neither ch14.18 IgG1 nor H3‐16 IgG1m4 mediated the lysis of U‐87 MG (Fig. 2D), which may have been due to the expression of complement inhibitory proteins (CD55 and CD59) on the cell surface.

**Fig. 3:**
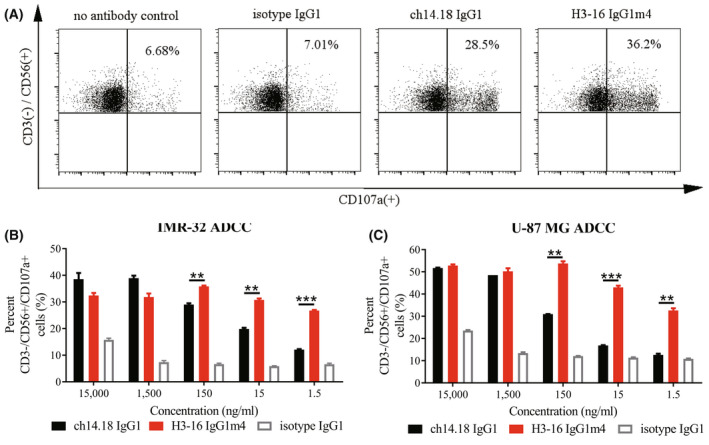
Extent of NK cell activation in ADCC mediated by H3‐16 IgG1m4. (A) A single representative subject of flow cytometry images showed the proportion of CD107a^+^ cells in the CD3^−^/CD56^+^ cell population in ADCC experiments with IMR‐32 as target cells. CD3^−^/CD56^+^/CD107a^+^ cells are the activated NK cell population. Ch14.18 IgG1, H3‐16 IgG1m4, and isotype IgG1 were all 0.15 μg·mL^−1^, and the group without antibody was used as a negative control. Using IMR‐32 (B) or U‐87 MG (C) as the target cells of ADCC, and the proportion of CD3^−^/CD56^+^/CD107a^+^ cells in the CD3^−^/CD56^+^ cell population, was detected by flow cytometry in the presence of different concentrations (0.0015–15 μg·mL^−1^) of anti‐GD2 antibody. The isotype IgG1 antibody served as a IgG1 isotype control. Data are presented as mean ± SEM (*n* = 3) and evaluated using an unpaired Student's *t*‐test (*P < 0.05, **P < 0.01, ***P < 0.001, and ****P < 0.0001). [Colour figure can be viewed at wileyonlinelibrary.com]

We also compared the potency of the anti‐GD2 antibodies in mediating the lysis of IMR‐32 cells using PBMCs from healthy donors. Ch14.18 IgG1 was more stable in mediating the lysis of the IMR‐32 cell line by PBMCs in the presence of a high concentration of antibodies (10–100 nm). Since the antibody concentration was reduced (equal to or lower than 1 nm), H3‐16 IgG1m4 was superior to ch14.18 IgG1 in the lysis ability (Fig. 2E). Similar results were obtained when the NB cell line, LAN‐5, was used as the target cells (data not shown). However, H3‐16 IgG1m4 showed stronger ADCC effects than ch14.18 IgG1 at all tested concentrations (Fig. 2F) when the U87‐MG cells with low GD2 expression were used as the target cells. The proportion of CD107a‐positive cells in the CD3^−^/CD56^+^ cell population was proportional to the extent of target cell lysis in ADCC (Fig. 3A–C), suggesting that the enhanced antitumor activity of the H3‐16 IgG1m4 antibody was associated with its enhanced ability to activate NK cells.

The binding affinities of the WT IgG1 and mutants to human recombinant FcγRs were compared using BLI, and the data are shown in Table 1. H3‐16 IgG1 and H3‐16 IgG1m4 both had higher binding affinities for CD16A‐158V than CD16A‐158F and higher KD for CD32A‐131H than CD32A‐131R. H3‐16 IgG1m4 had affinity toward the high‐ or low‐affinity allotypes CD16A and CD32A, similar to that of the parent H3‐16 IgG1. The above results concluded that compared with ch14.18 IgG1, H3‐16 IgG1m4 reduced the CDC effect and enhanced ADCC activity at low concentrations.

**Table 1:** Affinities of the H3‐16 Fc mutant antibodies binding to human FcγR allotypes.

FcγR	Antibody	kon (1/Ms)	kdis (1/s)	KD (M)
CD16A‐158V	H3‐16 IgG1	1.08E+5	7.99E‐2	7.38E‐7
	H3‐16 IgG1m4	8.64E+4	4.19E‐2	4.85E‐7
CD16A‐158F	H3‐16 IgG1	8.04E+4	2.98E‐1	3.70E‐6
	H3‐16 IgG1m4	5.92E+4	3.15E‐1	5.33E‐6
CD32A‐131R	H3‐16 IgG1	7.54E+5	1.31E‐2	1.74E‐8
	H3‐16 IgG1m4	1.59E+6	3.01E‐2	1.89E‐8
CD32A‐131H	H3‐16 IgG1	1.14E+6	1.33E‐2	1.16E‐8
	H3‐16 IgG1m4	1.35E+6	1.43E‐2	1.06E‐8

### H3‐16 IgG1m4 reduced allodynia in the SD rats

**Fig. 4:**
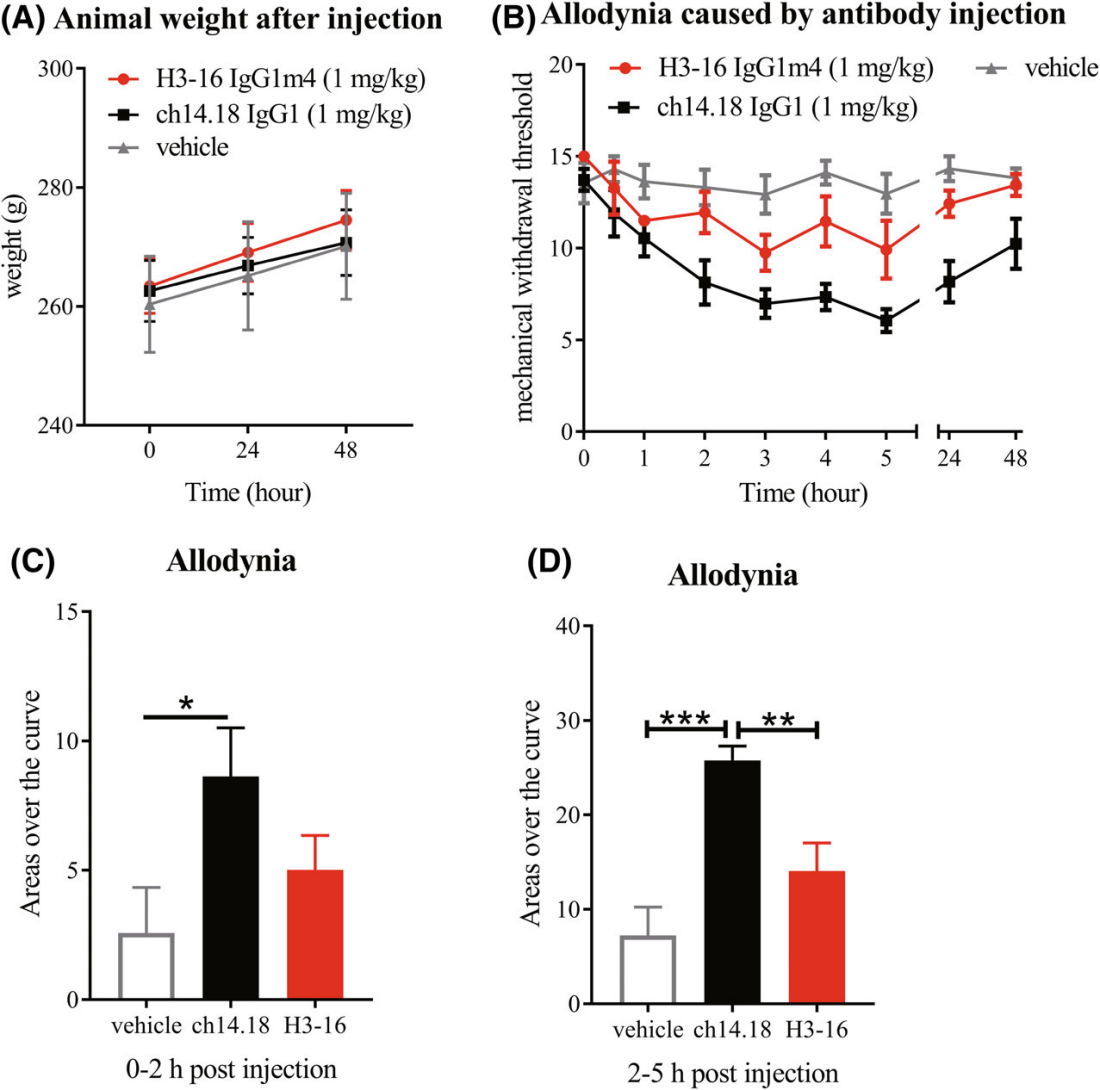
H3‐16 IgG1m4 injection‐induced changes in the mechanical paw withdrawal threshold in the Sprague–Dawley (SD) rats. (A) Animals' body weights were assessed prior to injection and at 24 and 48 h after injection. (B) SD rats were randomly divided into three groups, and the same volume of H3‐16 IgG1m4, ch14.18 IgG1, or PBS was injected into the tail vein. At the same time interval after injection, von Frey was used to detect the rats' paw contraction reaction, and the mean mechanical withdrawal threshold was calculated. The lower the mean mechanical withdrawal threshold of the rats, the stronger the pain side effects caused by the anti‐GD2 antibody injection. The curve of thresholds of animals injected with PBS is shown for a negative baseline. (C, D) Areas over the curve of the first 2 h after injection and 2–5 h after injection; the increasing numbers indicate that the rats had strong allodynia. Data are presented as mean ± SEM (*n* = 6) and evaluated using an unpaired Student's *t*‐test (*P < 0.05, **P < 0.01, and ***P < 0.001). [Colour figure can be viewed at wileyonlinelibrary.com]

Rats were divided into three groups using the random block method according to their body weight and mean mechanical withdrawal thresholds. There was no significant change in the body weight of the three groups during the experiment (Fig. 4A). After intravenous injection of the PBS buffer (vehicle group), the thresholds had little change in the next 48 h (Fig. 4B). After treatments with the anti‐GD2 antibodies (1 mg·kg^−1^, i.v., one dose), the thresholds of the rats were decreased (Fig. 4C,D). Two hours postinjection of ch14.18 IgG1, the threshold was 8.14 ± 1.21 g, significantly lower than that of the vehicle group (*P* < 0.05). The mean mechanical withdrawal thresholds were also lowered by the H3‐16 IgG1m4 treatment, but there was no statistical significance when compared to the vehicle group (*P* > 0.05). Thus, H3‐16 IgG1m4 attenuated allodynia in the SD rats when compared to ch14.18 IgG1.

## Discussion

GD2‐specific antibodies can prolong the EFS rate and overall survival rate of NB patients [16, 18, 28, 29], but have common side effects of neuropathic pain and antidrug antibody (ADA) reactions. Several anti‐GD2 antibodies, such as HM3F8 [30], 8B6 [31, 32], hu14.18K322A, and hu3F8, are being developed to reduce the side effects. Among them, hu14.18K322A and hu3F8 are generated by antibody engineering, and clinical trials have shown that they reduced pain and the ADA reaction [16, 17, 29]. In order to obtain a novel GD2‐specific antibody with even lower pain and a stronger antitumor capability, we made a series of modifications to ch14.18 IgG1 and obtained H3‐16 IgG1m4. The efficacy and side effects of the modified antibody H3‐16 IgG1m4 were evaluated *in vitro* and *in vivo*.

CDC is considered to be the primary cause of neuropathic pain in humans following anti‐GD2 antibody administration [20]. The humanized antibody, hu14.18K322A, has significantly reduced CDC activity *in vitro* [16, 17, 20] and was shown to have less neuropathic pain in a clinical trial [33] when compared to ch14.18 [18]. The K322A mutation is part of the Fc modification of H3‐16 IgG1m4. Similar to hu14.18K322A, H3‐16 IgG1m4 had reduced CDC effects *in vitro* and ameliorated neuropathic pain responses in rats, although there is no clinical evidence as yet.

Another aim of Fc modification is to improve the ADCC effects, which are considered the most important antitumor mechanisms of GD2‐specific antibodies. It was reported previously that the efficacy of anti‐GD2 antibodies in an EL4 syngeneic mouse model of metastatic lymphoma was only slightly reduced in mice deficient in C3 or the complement receptor 3 (CR3), but was nearly absent in mice deficient in FcγR I/III [13]. After the CDC activities of the anti‐GD2 antibodies were weakened, their ADCC activity should have been enhanced to compensate for their curative effects. Hu14.18K322A had reduced CDC activity but maintained ADCC activity because the fucose level of the antibody produced by the YB2/0 cell production system was lower than that in the SP2/0 cells. However, hu14.18K322A antibodies produced in different laboratories showed different ADCC effects. Federico et al. [34] tested six batches of hu14.18K322A produced according to the Current Good Manufacture Practices (cGMP) standards (produced by YB2/0 cells) and two clinical batches of dinutuximab (ch14.18). They found that the *EC*_*50*_ value of hu14.18K322A for the cleavage of GD2‐expressing cells was consistently 3.5–4 times lower than that of ch14.18. Sorkin et al. [20] reported that their laboratory transfected YB2/0 cells to express hu14.18K322A, whose ADCC results were only slightly lower than that of ch14.18 IgG1. We tried a different strategy to enhance the ADCC activity of the GD2 antibodies by introducing K322A, L235V, F243L, R292P, Y300L, and P396L mutations together into the Fc protein. The combination of mutations had been applied to the anti‐HER2 antibody, margetuximab, and was validated in clinical trials before [35]. We used BLI to measure the interaction between the Fc fragment and the IgG Fc receptors. The binding affinities of the mutant IgG1m4 to FcγII R and FcγIII R were similar to that of the nonmutant IgG1. The results indicated that, compared to WT IgG1, IgG1m4 might have similar activity for mediating granulocytes or NK cells to lyse tumor cells.

The antibody–antigen binding affinity is one of the factors that affects the ADCC activity [36], in addition to the Fc effector functions. Our results showed that, compared with ch14.18 IgG1, H3‐16 IgG1m4 had significantly increased binding activity to purified GD2 molecules and GD2‐positive cells. Furthermore, the cross‐binding activity of H3‐16 IgG1m4 with other gangliosides was similar to that of ch14.18 IgG1. After affinity maturation, the ADCC activity of H3‐16 IgG1m4 was enhanced. In order to lyse IMR‐32 and U‐87 MG cells *in vitro*, H3‐16 IgG1m4 required only a low concentration of antibody to achieve ADCC effects similar to those of a high concentration of ch14.18 IgG1.

Mouse xenograft tumor models are often used to evaluate the antitumor activity of antibodies *in vivo*. However, the primary antitumor mechanism of anti‐GD2 antibodies is its ADCC effect, and this requires the participation of activated receptors on the immune effector cells, such as human CD16A on the surface of NK cells. The distribution of murine FcγRs on mouse effector cells differs from their human counterparts, and the binding affinity of human IgG1 to murine FcγRs differs from that of human FcγRs [21]. Particular caution should be taken when evaluating the *in vivo* antitumor activity of antibodies improved for human FcγRs interaction. MGAH22 is an antibody that targets HER2 of the IgG1 subtype, and its Fc has been modified to enhance the activity of binding human CD16A. Nordstrom et al. [19] observed the superior antitumor activity of MGAH22 over WT Fc antibodies only in mCD16^−/−^ hCD16A^+^ mouse xenograft models, but not in FcγR‐WT mice or mCD16^−/−^ mice models. Constructing a scientific mouse xenograft model and evaluating the antitumor activity of Fc‐modified H3‐16 IgG1m4 *in vivo* will be the focus of our future research.

Ch14.18 IgG1 or H3‐16 IgG1m4 failed to trigger the CDC effect against U‐87 MG cells, which may have been due to the expression of CD55 and CD59 on their surfaces (www.proteinatlas.org). CD59 renders tumor cells resistant to CDC but does not affect ADCC [13]. The deficiency of CD55 or CD59 on the surface of the NB cells makes them more susceptible to CDC lysis. Nearly all human tumor cells, except NB cells, express CD59 [13]; thus, enhanced ADCC activity of H3‐16 IgG1m4 might be more important in GD2‐positive cancers other than NB [12]. Reduced CDC activity and painful side effects would facilitate expanding its scope of clinical application.

The clinical trials in NB patients have demonstrated that 43% or more of patients had ADA reactions against 14.G2a [9, 37], 19–21% against ch14.18 [38], and 40% against humanized antibody hu14.18K322A [16]. ADA would lead to low serum antibody levels and diminished therapeutic effects during subsequent treatment cycles [38]. The murine GD2 antibody 3F8 induced ADA in 70–80% of patients [39, 40, 41], but the humanized antibody, hu3F8, only induced ADA in 21% of patients [16, 42]. We humanized ch14.18 IgG1 with the framework shuffling technique based on the combination of the phage display library and high‐throughput screening. Online tools for antibody humanization, such as H‐score, G‐score, and T20, have been used for antibody screening and design [43, 44, 45], but *in vivo* evaluation is critical [36] and ongoing.

## Conclusion

Monoclonal antibody targeting disialoganglioside GD2 is an important therapeutical option for high‐risk neuroblastoma. However, anti‐GD2 antibodies can cause severe neuropathic pain, and this has limited their clinical application. By utilizing antibody engineering, we generated a humanized anti‐GD2 mAb H3‐16 IgG1m4 with a high affinity for GD2. Compared to its parental mAb ch14.18 IgG1, H3‐16 IgG1m4 showed enhanced ADCC and decreased CDC *in vitro*. The neuropathic pain effects of H3‐16 IgG1m4 were also significantly reduced *in vivo*. Taken together, our data demonstrated that H3‐16 IgG1m4 is a promising preclinical candidate for NB and other GD2‐positive tumors.

## Conflict of interest

Y‐LC, X‐YL, X‐ND, YC, X‐CD, H‐YL, and JT received stipends from Shanghai Mabstone Biotechnology, Ltd. G‐JL, G‐JC, and C‐HW are employees of Dartsbio Pharmaceuticals, Ltd.

## Author contributions

Conceptualization, Y‐LC and C‐HW; methodology, Y‐LC; validation, X‐YL, G‐JL, X‐ND, YC, X‐CD, H‐YL, JT, and G‐JC; formal analysis, Y‐LC, X‐YL, and C‐HW; investigation, Y‐LC and C‐HW; resources, Y‐LC, C‐HW, and Z‐MO; data curation, Y‐LC and X‐YL; writing, original draft preparation, X‐YL; writing, review, and editing, Y‐LC, Z‐MO, and C‐HW; visualization, X‐YL, X‐ND, and YC; project administration, Y‐LC; funding acquisition, C‐HW. All authors have read and agreed to the published version of the article.

## Data Availability

The data presented in this study are available from the corresponding author on reasonable request.
